# Pattern and predictors of mortality in necrotizing fasciitis patients in a single tertiary hospital

**DOI:** 10.1186/s13017-016-0097-y

**Published:** 2016-08-08

**Authors:** Gaby Jabbour, Ayman El-Menyar, Ruben Peralta, Nissar Shaikh, Husham Abdelrahman, Insolvisagan Natesa Mudali, Mohamed Ellabib, Hassan Al-Thani

**Affiliations:** 1 Department of Surgery, Hamad General Hospital (HGH), Doha, Qatar; 2 Clinical Research, Trauma Surgery, Hamad General Hospital, Doha, Qatar; 3 Clinical Medicine, Weill Cornell Medical School, Doha, Qatar; 4 Trauma Surgery Section, Hamad General Hospital, Doha, Qatar; 5 Surgical Intensive Care Unit, HGH, Doha, Qatar

**Keywords:** Necrotizing fasciitis, Predisposing factors, Presentation, Management, Mortality

## Abstract

**Background:**

Necrotizing fasciitis (NF) is a fatal aggressive infectious disease. We aimed to assess the major contributing factors of mortality in NF patients.

**Methods:**

A retrospective study was conducted at a single surgical intensive care unit between 2000 and 2013. Patients were categorized into 2 groups based on their in-hospital outcome (survivors versus non-survivors).

**Results:**

During a14-year period, 331 NF patients were admitted with a mean age of 50.8 ± 15.4 years and 74 % of them were males Non-survivors (26 %) were 14.5 years older (*p* = 0.001) and had lower frequency of pain (*p* = 0.01) and fever (*p* = 0.001) than survivors (74 %) at hospital presentation. Diabetes mellitus, hypertension, and coronary artery disease were more prevalent among non-survivors (*p* = 0.001). The 2 groups were comparable for the site of infection; except for sacral region that was more involved in non-survivors (*p* = 0.005). On admission, non-survivors had lower hemoglobin levels (*p* = 0.001), platelet count (*p* = 0.02), blood glucose levels (*p* = 0.07) and had higher serum creatinine (*p* = 0.001). Non-survivors had greater median LRINEC (Laboratory Risk Indicator for NECrotizing fasciitis score) and Sequential Organ Failure Assessment (SOFA) scores (*p* = 0.001). Polybacterial and monobacterial gram negative infections were more evident in non-survivors group. Monobacterial pseudomonas (*p* = 0.01) and proteus infections (*p* = 0.005) were reported more among non-survivors. The overall mortality was 26 % and the major causes of death were bacteremia, septic shock and multiorgan failure. Multivariate analysis showed that age and SOFA score were independent predictors of mortality in the entire study population.

**Conclusion:**

The mortality rate is quite high as one quarter of NF patients died during hospitalization. The present study highlights the clinical and laboratory characteristics and predictors of mortality in NF patients.

## Background

Necrotizing fasciitis (NF) is a rare infectious disease which is rapidly progressive and potentially fatal in nature [1]. Despite the advanced medical treatment, the rate of mortality remains as high as 24-34 %; posing a challenge for the diagnosis and management [2]. The mortality in NF patients primarily depends upon the time of the medical and surgical interventions and extent of spread of infection to the primary site (subcutaneous tissue, fasciae, skin or muscles) [3]. NF patients with streptococcal infection are associated with increased risk of complications and mortality (up to 80 %) [4]. There are various predisposing factors for NF such as advanced age, diabetes mellitus, peripheral vascular disease, obesity, chronic renal failure and trauma [5]. Therefore, early recognition of these predisposing factors might help in the early definitive management [6]. Moreover, early surgical debridement is a known contributor to improve outcomes in NF patients [5].

To date, the most appropriate tool for diagnosis and discrimination of NF is the LRINEC (Laboratory Risk Indicator for NECrotizing fasciitis score) scoring system proposed by Wong et al. [7]. It is based on laboratory parameters that are readily available for scoring in most institutions to predict survival and discriminate necrotizing from non-necrotizing infections. In addition, various predictors of mortality based on predisposing factors have been reported by different investigators. Previous studies have identified advanced age (>60 years), aeromonas and vibrio infection, liver cirrhosis, cancer, hypotension, band polymorphonuclear neutrophils (PMN) >10 %, and serum creatinine >2 mg/dL to be independent predictors of mortality in NF cases [8, 9]. Despite the fact that mortality depends upon the time of diagnosis, and management, the predisposing factors also play an important role in the outcome. The present study aims to determine the various predisposing and prognostic factors associated with mortality in NF patients.

## Methods

All consecutive patients admitted to the surgical intensive care at Hamad General Hospital (HGH) with a diagnosis of necrotizing fasciitis were retrospectively included in this analysis between January 2000 and December 2013. Patients were categorized into 2 groups according to their hospital mortality (survivors versus non-survivors). The present study included patients for which the operative notes and or histopathological findings indicate NF. Data included age, sex, presentation and duration of symptoms, predisposing factors, risk factors, causative microbiological organisms, on-admission laboratory parameters, the Sequential Organ Failure Assessment (SOFA) and LRINEC score, number of operative debridement, length of intensive care and hospital stay, recurrence and in-hospital mortality. The anatomic site of infection was classified as extremity (upper and lower limbs), abdominal/groin, chest/breast, neck/facial, sacral and perineum. Exclusion criteria included patients not admitted in the ICU (uncomplicated mild forms of NF and were managed in the regular ward), patients with cellulitis or superficial infection not requiring aggressive debridement or antibiotics), and non-surgical patients.

The ratio of partial pressure of arterial oxygen and fraction of inspired oxygen (PaO2/FiO2), platelets count, bilirubin level, Glasgow coma score, mean arterial pressure (MAP), vasopressor use, creatinine level and urine output were used to calculate SOFA score [10]. The laboratory parameters such as C-reactive protein (CRP), WBC, hemoglobin, sodium level, creatinine concentration and glucose level were used to calculate the LRINEC score [7]. The various recorded parameters were analyzed according to the final outcome. This study was approved by the medical research center at HMC, Qatar with IRB#14066/14.

### Statistical analysis

Data were presented as proportions, median (range) or mean (± standard deviation), as appropriate. Baseline demographic characteristics, laboratory findings, clinical presentation, bacteriology and predisposing factors were compared between non-survivors and survivors. Analyses were conducted using Student-t test for continuous variables and Pearson chi-square (χ^2^) test for categorical variables; Fisher’s exact test was used, if the expected cell frequencies were below 5. A 2-tailed *p* < 0.05 was considered significant. Multivariate logistic regression analysis was performed to look for the predictors of mortality in the overall NF cohort along with the odd ratio and 95 % confidence interval. Data analysis was carried out using the Statistical Package for Social Sciences version 18 (SPSS Inc, Chicago, Illinois).

## Results

During the 14-year study period, a total of 331 admissions were recorded for NF; 74 % were males and the mean age was 50.8 ± 15.4 years. Among them, 246 were survivors (74.3 %) and 85 (25.7 %) were non-survivors. Non-survivors were 14.5 years older (61.6 ± 14.3 vs. 47 ± 14 years, *p* = 0.001) than survivors and the two groups were comparable for gender. Moreover, higher proportion of Qatari nationals (50.6 % vs. 27.2 %; *p* = 0.001) died due to NF as compared to non-Qatari (Arabs) (Table 1).

**Table 1:** Comparison of necrotizing fasciitis by outcome (survivors versus non-survivors)

	All patients (*n* = 331)	Survivors (*n* = 246)	Non- survivors (*n* = 85)	P *
Males	246 (74.3 %)	75 %	73 %	0.73
Age in years^a^	50.8 ± 15.4	47 ± 14	61.6 ± 14.3	0.001
Nationality				
Qatari	110 (33.2 %)	27.2 %	50.6 %	0.001
Non-Qatari (Arabs)	73 (22.1 %)	20.3 %	27.1 %	
Others	148 (44.7)	52.4 %	22.4 %	
Symptoms				
Swelling	237 (78 %)	76.4 %	82.3 %	0.28
Pain/tenderness	208 (68.4 %)	72.4 %	57 %	0.01
Fever	203 (67 %)	73.3 %	48 %	0.001
Laboratory findings				
Hemoglobin (g/dl)^a^	11 ± 2.7	11.4 ± 2.7	10.1 ± 2.6	0.001
WBC(/μl)^a^	16.2 ± 8.6	16.4 ± 8.6	15.2 ± 8.6	0.28
Platelet count (/μl)^b^	269 ± 201	273 ± 141	230 ± 158	0.02
Sodium (mmol/l)^a^	133.5 ± 5.6	133.4 ± 5.4	133.9 ± 6	0.43
Serum Creatinine (μmol/l)^b^	97 (26–1263)	91 (26–1189)	135 (26–1263)	0.001
Serum Bilirubin(μmol/l)^b^	14.2 (3–381)	14 (3–233)	15 (4–381)	0.35
Serum Glucose^a^	12.0 ± 7.8	12.5 ± 8.4	10.7 ± 5.4	0.07
C-reactive protein^a^	221 ± 120	214 ± 120	232 ± 120	0.35
Procalcitonin (<24 h)^b^ ^,c^	10.5 (0.07-303)	3.3 (0.07-303)	9.8 (0.1-182)	0.28
Scoring				
SOFA^b^	9 (2–21)	9 (2–19)	12 (7–21)	0.001
LRINEC^b^	6 (1–13)	5 (1–13)	7 (2–13)	0.001
Site				
Lower limb/Thigh	175 (53 %)	53.3 %	51.8 %	0.81
Perineum	81 (25 %)	23.6 %	27 %	0.52
Abdominal/Groin	38 (11.5)	10.6 %	14 %	0.37
Upper Limb	13 (3.9 %)	4.1 %	3.5 %	0.82
Neck/Facial	21 (6.3 %)	7.3 %	3.5 %	0.21
Chest/Breast	8 (2.4 %)	2.8 %	1.2 %	0.38
Sacral	5 (1.5 %)	0.4 %	4.7 %	0.005
Gluteus	3 (0.9 %)	1.2 %	0 %	0.30
Histopathological confirmation	192 (58 %)	61 %	49.4 %	0.06
Morbidity				
Diabetes Mellitus	167 (52 %)	47 %	64 %	0.007
Renal impairment	49 (15.2 %)	10 %	30 %	0.001
Coronary Artery disease	46 (14.2 %)	11 %	25 %	0.001
Trauma	43 (15.5 %)	18.2 %	8 %	0.04
Number of debridement^b^	2 (1–8)	2 (1–7)	2 (1–8)	0.22
Combined antibiotics(>2)	94(33.6 %)	49.3 %	28.2 %	0.001
Septic shock	76 (27.8 %)	19 %	51.4 %	0.001
ICU stay in days^b^	5.5 (1–75)	5 (1–43)	9 (1–75)	0.002
Hospital stay in days^b^	16 (2–295)	15 (2–295)	20.5(2–273)	0.02

^*^ = survivors vs. non-survivors, ^a^ = values in (mean ± SD), ^b^ = values in median and (range), c=<0.5ng/l low risk and >2.0 ng/l high risk sepsis

### Clinical findings

On admission, the most common symptoms were local swelling (78 %), pain/tenderness (68 %) and fever (67 %). At presentation, non-survivors had significantly lower frequency of pain (57 % vs. 72 %; *p* = 0.01) and fever (48 % vs. 73 %; *p* = 0.001) than survivors. The frequency of diabetes mellitus (64 % vs. 47 %; *p* = 0.007), hypertension (53 % vs. 29 %; *p* = 0.001), renal impairment (30 % vs. 10 %; *p* = 0.001), coronary artery disease (25 % vs. 11 %; *p* = 0.001) and cerebrovascular accidents (8 % vs. 1 %; *p* = 0.001) were significantly higher among non-survivors as compared to survivor group. However, traumatic injuries (18 % vs. 8 %; *p* = 0.04) were observed more among survivors than non-survivors.

### Site of infection

The most frequent site of infection was lower limb/thigh (53 %) followed by perineum (25 %), abdominal/groin region (11.5 %) and neck/facial region (6.3 %). Although, the 2 groups were comparable for the site of infection; sacral region had significantly higher frequency in non-survivors (4.7 % vs. 0.4 %; *p* = 0.005) than survivors.

### Laboratory findings

The initial blood investigations such as hemoglobin, leukocyte count, serum sodium, bilirubin and C-reactive protein were comparable among survivors and non-survivors. However, non-survivors had lower levels of hemoglobin (10.1 ± 2.6 vs. 11.4 ± 2.7; *p* = 0.001), platelet count (230 ± 158 vs. 273 ± 141; *p* = 0.02), blood glucose levels (10.7 ± 5.4 vs. 12.5 ± 8.4; *p* = 0.07) and had higher serum creatinine [135 (26–1263) vs. 91 (26–1189); *p* = 0.001] as compared to survivors. The median procalcitonin levels were non-significantly higher in non-survivors [9.8 (0.1-182) vs. 3.3 (0.07-303); *p* = 0.28] than that of survivors. In addition, non-survivors had significantly higher median LRINEC [7 (2–13) vs. 5 (1–13); *p* = 0.001] and SOFA scores [12 (7–21) vs. 9 (2–19); *p* = 0.001] in comparison to the survivors group. Also, non-survivors were less likely to receive combination of antibiotics (>2 antibiotics) than survivors (28.2 % vs. 49.3 %; *p* = 0.001).

#### Microbiological findings

Table 2 represents the involvement of microorganisms in the pathogenesis of NF. Monobacterial gram positive (42 %) were the most frequent organisms identified followed by polybacterial (34 %) and monobacterial gram negative (12.5 %). Among gram positive bacteria, streptococcus (38 %) and staphylococcus (37 %) were the most commonly identified organisms. Bacteriodes (22 %) and E-Coli (11 %) were the predominant gram negative microorganisms. Fungal infection was observed in 30 (10.2 %) cases. Among them 22 (73.3 %) cases were positive for tissue culture, 7 (23.3 %) were positive for tissue as well as blood culture and one (3.4 %) case was positive for blood culture alone. The frequency of polybacterial (38 % vs. 32.5 %, *p* = 0.002) and monobacterial gram negative infections (15.5 % vs. 11.3 %, *p* = 0.002) were more evident in non-survivors; while monobacterial gram positive organisms were commonly identified among survivors (47.9 % vs. 25.4 %; *p* = 0.002) compared to non-survivors.

**Table 2:** Micro-organisms involved in necrotizing fasciitis

	Overall	Survivors	Non-survivors	*P* value
Positive wound culture	204 (77 %)	80.4 %	67.6 %	0.03
Positive blood and tissue culture	56(21 %)	18 %	29.6 %	0.04
Polybacterial infection	90 (34 %)	32.5 %	38.0 %	0.002 for all
Monobacterial Gram positive	111 (42 %)	47.9 %	25.4 %	
Monobacterial Gram negative	33 (12.5 %)	11.3 %	15.5 %	
Fungal	30 (10.2 %)	6.9 %	19.2 %	
Gram positive				
Streptococcus	114 (38 %)	42 %	29 %	0.05
Staphylococcus	109 (37 %)	39 %	29 %	0.11
Enterococcus	14 (5 %)	4.5 %	5.3 %	0.78
Clostridium	3 (1 %)	1.4 %	0 %	0.30
Gram negative				
Bacteroides	61 (22 %)	20 %	22.4 %	0.64
E. Coli	34 (11 %)	10 %	16 %	0.16
Pseudomonas	23 (8 %)	5.4 %	14.5 %	0.01
Klebsiella	23 (8 %)	6 %	12 %	0.12
Aeromonas	4 (1.3 %)	0.9 %	2.6 %	0.26
Proteus	5 (1.7 %)	0.5 %	5.3 %	0.005
Morganella	2 (0.7 %)	0.5 %	1.3 %	0.42

Pseudomonas (14.5 % vs. 5.4 %; *p* = 0.01) and Proteus infections (5.3 % vs. 0.5 %; *p* = 0.005) were the most commonly associated microorganisms among non-survivors.

#### Management and outcomes

The median number of surgical debridements performed was 2 (ranged 1–8) and the hospital length of stay was 16 (2–295) days. The number of debridements was comparable in the 2 groups. The median ICU stay [9 (1–75) vs. 5 days (1–43); *p* = 0.002], overall hospital stay [20.5 (2–273) vs. 15 (2–295) days; *p* = 0.02] and the frequency of septic shock (48 % vs. 20 %; *p* = 0.001) were significantly higher in non-survivors than the survivor group. Recurrent admissions for NF were required for 13 (4 %) patients; of whom 11 patients were admitted twice and two patients required three admissions. A total of 85 patients died in the present study with an overall mortality rate of 26 %. Table 3 shows the major causes of mortality which mainly involved septic shock alone and a combination of bacteremia and multiorgan failure.

**Table 3:** Major causes of mortality (*n* = 85) in necrotizing fasciitis patients

Variable	Number
- Septic shock	22
- Bacteremia & multiorgan failure	25
- End stage renal disease and sepsis	2
- sepsis and Cardiopulmonary arrest	3
- Disseminated intravascular coagulation and sepsis	1
- Pulmonary embolism and sepsis	1
- Acute respiratory distress syndrome	2
- Stomach cancer and sepsis	1
- Encephalopathy and sepsis	1
- Volume overload/HF and sepsis	1
- Necrotizing pancreatitis + multiorgan failure	1
- Hypoxic brain injury and sepsis	1
- Myocardial infarction and sepsis	1
Septic myocarditis	1
Cardiac arrest	3
Cardiogenic shock	2
Myocardial infarction	1
Pneumonia	2
Acute myeloid leukemia and sepsis	1
Acute pulmonary edema	1
Missing/not defined	12

Table 4 shows multivariate analysis for the major predictors of mortality. SOFA scoring followed by age were the independent predictors of mortality in the present study cohort. The proportion of mortality based on the bacteriology results is given in Table 5.

**Table 4:** Multivariate analysis for predictors of mortality

	*P* value	Odd ratio	95 % confidence interval	
Gender	0.928	0.952	0.328	2.762
Age	0.001	1.06	1.03	1.11
Serum hemoglobin	0.416	1.088	0.888	1.333
Serum sodium	0.442	0.965	0.883	1.056
Serum glucose	0.887	0.995	0.926	1.069
Serum creatinine	0.557	0.999	0.997	1.002
SOFA score	0.020	1.23	1.03	1.49
Lower Limb NF	0.979	1.017	0.278	3.719
Perineum NF	0.891	1.096	0.296	4.059
Abdominal NF	0.671	1.496	0.234	9.574
Prior coronary artery disease	0.917	1.060	0.355	3.162
Monobacterial Gram positive	0.086	0.435	0.168	1.124

*NF* necrotizing fasciitis

**Table 5:** Mortality based on the bacteriology results

	Number of cases	Mortality
Polybacterial infection	90	27 (38 %)
Gram Positive alone	111	18 (25.4 %)
Gram negative alone	33	11 (15.5 %)
Fungal^a^	30	15 (21.1 %)
Total^b^	265	71

^a^overlap with Gram stain bacteria, ^b^Confirmed results

None of the co-morbidities showed significant association with types of microorganisms and combination of antibiotics used except coronary heart disease (CHD). Significantly higher frequency of CHD patients were prescribed more than two antibiotic combinations (*p* = 0.009). Table 6 compares the co-morbidities with microbiological data and antibiotics used.

**Table 6:** Comparison of co-morbidities with microbiological data and antibiotics

	Diabetes mellitus (*n* = 167)	Renal impairment (*n* = 49)	Coronary artery disease (*n* = 46)	Trauma (*n* = 43)
Polybacterial infection	51 (30.5 %)	14 (28.6 %)	10 (21.7 %)	18 (41.9 %)
Monobacterial Gram positive	54 (32.3 %)	12 (24.5 %)	11 (23.9 %)	16 (37.2 %)
Monobacterial Gram negative	19 (11.4 %)	7 (14.3 %)	6 (13.0 %)	0 (0.0 %)
Fungal	21 (12.6 %)	9 (18.4 %)	7 (15.2 %)	2 (4.6 %)
Antibiotic combination used (>2)	47 (28.1 %)	18 (36.7 %)	21 (45.6 %)^a^	13 (30.2 %)

^a^ statistically significant

## Discussion

The association of high morbidity and mortality in necrotizing fasciitis (NF) patients urges the need for early diagnosis and identification of potential risk factors of worse outcomes. The present study is interestingly large series of NF cases from a Middle Eastern small population country that assesses various contributing factors to mortality. NF is a fulminant life-threatening infection of the musculoskeletal soft tissues characterized with rapid progression that typically requires urgent surgical interventions [11, 12]. The classic and frequent manifestations associated with NF usually include a triad of pain, tender local swelling, and fever [13, 14]. Consistent with earlier reports, this triad was more frequently observed among survivors in the current series. Moreover, out of proportion pain on physical examination and unresolved cellulitis are major diagnostic clues for NF, however, these clinical features often appear later in the disease course [9]. Therefore, delayed diagnosis is usually associated with high mortality (up to 25 %) among young adults which could even reach 44 % in elderly population [9, 15]. The number of in-hospital deaths in the present study is 26 % which is consistent with earlier studies.

The current literature suggests that NF could occur at any age but is mostly reported within the age range of 32 to 57 years [16, 17]. In the present study, the mean age was 51 years and the non-survivors were 14.5 years older than the survivors at the time of presentation. The reason of the frequently observed association of NF with advanced age could be explained in part by the pre-existing co-morbidities and immunosuppression. In this context, Golger et al. [18] reported advanced age, streptococcal toxic shock syndrome and immunocompromised status to be independent predictors of mortality in NF patients.

The frequently associated co-morbidities in NF are diabetes mellitus, malignancy, chronic cardiac disease, peripheral vascular disease, chronic renal disease, and immune-suppression [19]. Other predisposing factors include traumatic injuries, smoking, history of muscular injection and paraplegia. Diabetes mellitus, hypertension, and renal impairment were the most frequent co-morbidities associated with mortality in the current series. Diabetes mellitus remains the main co-morbidity in NF patients which is associated with prolonged hospitalization and increased mortality [13, 20]. In this study, patients with a history of diabetes mellitus showed considerably rapid progress of the severity of NF and mortality. This finding could be attributed in part to the hyperglycemic status that compromises the immunity status and fosters bacterial growth. However, the initial readings of serum sugar in the study cohort were non-significantly lower in non-survivors. Unfortunately, the current database did not include HbA1c to explain in-part this finding. The other common comorbidity in the present study was hypertension, which might cause disruption of the microvascular supply and reduction of tissue oxygenation and antimicrobial delivery. The frequency of hypertension was significantly higher in the non-survivors group. Consistently, Huang et al. [8] demonstrated a high association of hypertension among NF non-survivors. Earlier studies have also outlined the increased risk of NF in the presence of the above-mentioned pathologies [21, 22]. Furthermore, elderly patients with such co-morbidities who are suspected to have NF should be evaluated thoroughly to rule out NF, even in the absence of the usual hard manifestations.

Although, NF might affect any part of the body, earlier studies have reported frequent involvement of the extremities, perineum, head & neck and truncal regions [23]. In the current series, the most frequent sites of infection included lower limbs, perineum, abdominal/groin and neck/facial regions. The site of infection and its expansion also affect mortality. It has been suggested that affection of the head and neck region is associated with higher mortality as accounted for the proximity with various vital anatomical structures [24]. Mao et al. [25] analyzed the craniocervical NF cases with and without thoracic extension and observed a poor survival with thoracic extension as compared to non-thoracic extension. An earlier study reported a lower rate of mortality in extremity infection in comparison to abdominal and perineal infections [26]. Urschel [27] suggested that NF infection extending proximally to pelvis or trunk might have worse prognosis. Therefore, early and aggressive treatment aimed at restriction of the infection with repeated surgical debridement could be useful in achieving better survival rates.

Unfortunately, the first stage of NF disease is frequently masked by non-specific manifestations, which prevents effective and timely specific therapy [28]. Therefore, early identification and diagnosis is mandatory and should not rely only on the clinical signs alone [6]. Consequently, prognostic indicators such as laboratory markers and specific patient characteristics obtained from the medical history would assist in the early diagnosis, risk stratification and decision making [29]. Earlier studies identified some laboratory findings such as anemia, elevated creatinine, and increased white cell count to be non-specifically associated with NF which might affect prognosis. In the present series, non-survivors had significantly lower levels of hemoglobin and platelet count and had higher serum creatinine as compared to survivors. Similarly, an earlier study observed that non-survivors had significantly lower levels of hemoglobin and platelet and presented with higher levels of serum glucose and creatinine than the survivors [8]. An earlier study reported that aeromonas infection, advanced age, band PMNs >10 %, serum creatinine (>2.0 mg/dL), and an activated prothrombin time (>60 s) were found to be the independent predictors of mortality in NF patients [8]. In the present study the major predictors of mortality were age and SOFA scoring. SOFA score is a useful tool to assess the severity of NF based on the involvement of major organ systems. In the present series, non-survivors had significantly higher median SOFA scores which are in accordance with the current literature. The initial increase in SOFA score during the first two days of ICU admission successfully predicts high rates of mortality (50-95 %) [30]. This finding could be used as an alarming indictor and encourages physicians to refer those patients as early as possible to the tertiary care centers for the appropriate intensive care. Therefore, the use of validated prognostic factors in daily clinical practice, especially for initial diagnosis in emergency departments, would help physicians for timely management and obtaining better outcomes.

It has been suggested that bacteremia is one of the frequent complications of NF which has been associated with higher risk of mortality [31]. In the current study, 21 % of the patients had positive blood and tissue cultures and subsequently had higher mortality rate in comparison to those who had negative blood culture. Similarly, Huang et al. [8] observed four-fold increased rate of mortality in patients with positive blood cultures than those who had negative cultures.

Consistent with previous reports [13], gram positive microorganisms, mainly streptococcus and staphylococcus organism, were frequently identified in the present study cohort. On the other side, bacteriodes and E-coli were the predominant gram negative organisms. In the present study, monobacterial infections with pseudomonas and proteus were the most commonly associated microorganisms among non-survivors. However, earlier studies reported clostridial [32], beta-streptococci [33], aeromonas and vibrio [9] infections to be associated with poor outcomes.

Prompt and aggressive debridement is important for the management of NF. The debridement aims to remove all necrotic tissue until the local infectious process is treated. There is a positive correlation between the survival rate and early diagnosis with appropriate surgical debridement in NF patients [34]. In the present study, the median number of debridement procedures performed per patient was two interventions, and these were comparable for both non-survivors as well as survivors. Data suggested that early surgical intervention is crucial in reducing morbidity and mortality in NF patients [35]. However, there is still a lack of clear definition on ‘How early should we be’. Kobayashi et al. showed significantly lower mortality in the early intervention group (within 12 h after diagnosis) [36]. Delay of surgical treatment of >12 h was associated with an increased number of surgical debridement, septic shock and acute renal failure [36]. Hadeed et al. [35] reported outcomes of earlier surgical treatment (within the first 6 h) and found that although there was no statistically significantly difference in mortality between the study groups, higher mortality among late intervention group was clinically significant. Moreover, the outcomes in terms of the duration of hospital and intensive care unit stay were in favor of early intervention [35].

The appropriate and early antibiotic use and intensive care measures significantly appear to affect patients’ outcomes. In the past decades, patients with NF have higher mortality rates (up to 70 %), however, currently with improved surgical and intensive care treatment, mortality rates have declined to less than 30 % [37, 38]. Not only delayed diagnosis and surgical intervention influences in-hospital mortality, but also, the development of secondary complications has unfavorable impact [39]. The major complications that significantly related to mortality in the present series were bacteremia, septic shock and multiorgan failure. Therefore, appropriate prevention and management of such complications are vital for improving the outcome in these vulnerable patients [39].

Table 7 summarizes the published studies of mortality in NF and NSTI worldwide between 2000 and 2016. There is no consensus for specific predictors of mortality between these 20 studies including the current study. The design and objectives of studies as well as the availability of clinical and laboratory data are the main reason of the diversity of predictors of mortality among these studies. The present study has several limitations. It is retrospective in nature. It lacks information regarding the exact time of commencing antibiotics, delay in diagnosis, the timing of surgical debridement and the type of surgery performed post diagnosis. Also, the sensitivity and minimum inhibitory concentration of the bacteria and percentage of multi drug resistant strains is not available . Data describing the empiric antibiotic treatment in the emergency room is also not available for analysis. Moreover, procalcitonin has been introduced recently at HGH; therefore not all the NF in the past underwent procalcitonin assessment at admission. Further prospective studies are required to determine the time interval between the diagnosis and treatment which could possibly influence the mortality among NF patients.

**Table 7:** Summary of published studies of mortality in necrotizing fasciitis/NSTI patients worldwide

Authors	Year/country	Study type/duration	Mortality	Predictors of mortality
Dahm P et al. [40]	2000/USA	Retrospective/1984 to 1998	Overall mortality rate was 20 % (10/50)	The extent of the infection (*P* = 0.0234) was the only significant, independent predictor of outcome
Chin-Ho Wong et al. [29]	2003/Singapore	Retrospective/1997 to 2002	Total *n* = 89	A delay in surgery of > 24 h was correlated with increased mortality (*p* < 0.05; RR = 9.4)
Daniel A. Anaya et al. [32]	2005/USA	Retrospective/1996 to 2001	The overall mortality rate was 16.9 % (total *n* = 166)	Independent predictors of mortality included WBC > 30 000 × 103/μL, creatinine level > 2 mg/dL (176.8 μmol/L), and heart disease at hospital admission
Kwan MK et al. [41]	2006/Malaysia	Retrospective/1998 to 2002	Overall mortality rate was 36 % (total *n* = 36)	A poor WBC response, high serum urea and creatinine, and low haemoglobin level were the predictors for mortality
Golger A et al. [18]	2007/Canada	Retrospective/1994 to 2001	Ninety-nine patients satisfied the inclusion criteria. Overall mortality was 20 %	Advanced age (OR, 1.04; 95 % CI, 1.01 to 1.08; *p* = 0.012), streptococcal toxic shock syndrome (OR, 10.54; 95 % CI, 2.80 to 39.44; *p* < 0.001), and immunocompromised status (OR, 3.97; 95 % CI, 1.04 to 15.19; *p* = 0.044) were independent predictors of mortality
Mulla ZD et al. [42]	2007/USA	Case series/2001	The crude hospital mortality rate was 11.1 % (total *n* = 216)	Patients aged > or =44 years at the time of admission were 5 times as likely to die in the hospital than patients who were aged < or =43 years (adjusted RR 5.08, *P* = 0.03)
Hsiao CT et al. [9]	2008/Taiwan	Retrospective/2002 to 2005	^a^24/128 (19 %)	Aeromonas infection, Vibrio infection, cancer, hypotension, and band form WBC > 10 % were independent positive predictors of mortality (*P* < 0.05). Presence of hemorrhagic bullae was a negative predictor of mortality (*P* < 0.05)
Bair MJ et al. [43]	2009/Taiwan	Retrospective/1995 to 2006	The overall mortality was 17.0 %. total *n* = 85	Predictors of mortality included advanced age, class C liver cirrhosis, ascites, higher serum creatinine, and lower hemoglobin and platelet levels
Kuo Chou TN et al. [44]	2010/Taiwan	Retrospective/2000 to 2007	24/119 (20 %)	The presence of hemorrhagic bullous skin lesions/necrotizing fasciitis, primary septicemia, a greater severity of illness, absence of leukocytosis, and hypoalbuminemia were the significant risk factors for mortality
Kao LS et al. [45]	2011/USA	Retrospective/2004 to 2007	Mortality rates varied between 6 hospitals from 9 % to 25 % (*n* = 296)	Patient age and severity of disease (reflected by shock requiring vasopressors and renal failure postoperatively) were the main predictors of mortality
Huang KF et al. [8]	2011/Taiwan	Retrospective/2003 to 2009	Overall mortality was 12.1 % (*n* = 57/472) and the 30 day mortality was 11.0 % (*n* = 52/472)	Eight independent predictors of mortality : liver cirrhosis, soft tissue air, Aeromonas infection, age > 60 years, band polymorphonuclear neutrophils >10 %, activated partial thromboplastin time >60 s, bacteremia, and serum creatinine >2 mg/dL
Yeung YK et al. [46]	2011/Hong Kong	Retrospective	Overall mortality was 28 % (total *n* = 29)	Renal and liver failure, thrombocytopenia, initial proximal involvement, and hypotension on admission were predictors of mortality in UL NF. The ALERTS (Abnormal Liver function, Extent of infection, Renal impairment, Thrombocytopenia, and Shock) score with a cutoff of 3 appeared to predict mortality.
Nisbet M et al. [47]	2011/New Zealand	Retrospective/2000 to 2006	Twenty-five (30 %) patients died, 17 (68 %) within 72 h of admission. Total *n* = 82	Independent predictors of mortality include congestive heart failure (*P* = 0.033) and a history of gout (*P* = 0.037)
Krieg et al. [48]	2014/Germany	Retrospective/1996 to 2011	^a^24/64(32.8 %)	Independent predictors of mortality were skin necrosis on the initial clinical examination (OR = 15.48; 95 % CI = 2.02–118.91) and acute renal failure (OR = 118.91; 95 % CI 7.66–5135.79)
Lee YC et al. [49]	2014/Taiwan	Retrospective/1996 to 2011	18/100 (18 %)	Unknown injury events, presence of multiple skin lesions, leukocytes < 10,000 cells/mm^3^, platelets < 100,000/mm^3^, serum creatinine ≥1.3 mg/dL, serum albumin < 2.5 mg/dL, and delayed treatment beyond 3 days post-injury were associated with significantly higher mortality. Treatment delayed beyond 3 days is an independent factor indicating a poor prognosis (OR 10.75, 95 % CI 1.02-113.39, *p* = 0.048)
Khamnuan P et al. [50]	2015/Thailand	Retrospective/2009 to 2012	*n* = 290/1504 (19.3 %)	Female gender; age >60; chronic heart disease, cirrhosis, skin necrosis, pulse rate >130/min, systolic BP <90 mmHg, and serum creatinine ≥1.6 mg/dL
Khamnuan P et al. [51]	2015/Thailand	Retrospective observational cohort study/2009 to 2012	165 (69.6) in patients with severe sepsis (*n* = 237) 66 (5.5) without severe sepsis (*n* = 1,215) *P* <0.001	Female sex, diabetes mellitus, chronic heart disease, hemorrhagic bleb, skin necrosis, and serum protein <6 g/dL
Arif et al. [52]	2016/USA	Retrospective/2003 to 2013	9871 NF-related deaths 4 · 8 deaths/ 1000000 person-yr	Diabetes mellitus, obesity, and renal failure were significantly associated with NF-related death. However, age, sex, and race were independently associated with the rate of NF-related deaths
Hadeed GJ et al. [35]	2016/USA	Retrospective/2003 to 2008	11/87 (12.5 %)	Clinically significant difference based on the timing of surgical intervention (< or > 6 h) (17.5 % in late vs. 7.5 % in early intervention group), however no statistical significance

^a^ = Deaths/total NF cases

## Conclusion

The mortality rate is quite high as one quarter of NF patients died in the hospital. The present study highlights the clinical characteristics and predictors of mortality in NF patients. It is important to have a high index of suspicion at initial presentation. Use of prognostic tools in the daily clinical practice will help physicians for the proper on-time management. The present study provides useful information on the severity and outcome of NF patients that will inform institutional guidelines for the on-time treatment of NF.

## Abbreviations

CRP, C-reactive protein; LRINEC, laboratory risk indicator for necrotizing fasciitis score; MAP, mean arterial pressure; NF, necrotizing fasciitis; NSAID, non-steroidal anti-inflammatory drug; SOFA, sequential organ failure assessment.
